# Lactoferrin Promotes Early Neurodevelopment and Cognition in Postnatal Piglets by Upregulating the BDNF Signaling Pathway and Polysialylation

**DOI:** 10.1007/s12035-014-8856-9

**Published:** 2014-08-23

**Authors:** Yue Chen, Zhiqiang Zheng, Xi Zhu, Yujie Shi, Dandan Tian, Fengjuan Zhao, Ni Liu, Petra S. Hüppi, Frederic A. Troy, Bing Wang

**Affiliations:** 1 School of Medicine, Xiamen University, 361005 Xiamen, China; 2 Nestle Research Center, 100095 Beijing, China; 3 Division of Development and Growth, Department of Paediatrics, University of Geneva School of Medicine, 1211 Geneva, Switzerland; 4 Department of Biochemistry and Molecular Medicine, University of California School of Medicine, Davis, CA 95616 USA; 5 School of Animal & Veterinary Sciences, Charles Sturt University, Wagga Wagga, 2678 Australia

**Keywords:** Lactoferrin, BDNF signaling pathway, CREB, Polysialic acid-NCAMs, Neurodevelopment, Gene microarray and qPCR, Cognition

## Abstract

Lactoferrin (Lf) is a sialic acid (Sia)-rich, iron-binding milk glycoprotein that has multifunctional health benefits. Its potential role in neurodevelopment and cognition remains unknown. To test the hypothesis that Lf may function to improve neurodevelopment and cognition, the diet of postnatal piglets was supplemented with Lf from days 3 to 38. Expression levels of selected genes and their cognate protein profiles were quantitatively determined. The importance of our new findings is that Lf (1) upregulated several canonical signaling pathways associated with neurodevelopment and cognition; (2) influenced ~10 genes involved in the brain-derived neurotrophin factor (BDNF) signaling pathway in the hippocampus and upregulated the expression of polysialic acid, a marker of neuroplasticity, cell migration and differentiation of progenitor cells, and the growth and targeting of axons; (3) upregulated transcriptional and translational levels of BDNF and increased phosphorylation of the cyclic adenosine monophosphate (cAMP) response element-binding protein, CREB, a downstream target of the BDNF signaling pathway, and a protein of crucial importance in neurodevelopment and cognition; and (4) enhanced the cognitive function and learning of piglets when tested in an eight-arm radial maze. The finding that Lf can improve neural development and cognition in postnatal piglets has not been previously described.

**Electronic supplementary material:**

The online version of this article (doi:10.1007/s12035-014-8856-9) contains supplementary material, which is available to authorized users.

## Introduction

Infants are born with their neurons already formed. However, the synaptic connections between these neurons are in a large part established and elaborated after birth. The process of rapid neural growth places an unusually high demand on the intracellular pool of biochemical precursors and nutrients [1, 2]. Human milk, in contrast to infant formula, is a unique nutrient known to modulate a myriad of biological functions involved in regulating optimal growth of the brain of newborn infants [3]. Lactoferrin (Lf), a heavily sialylated, iron-binding glycoprotein constitutes a quarter of the total protein in human milk [4]. Its concentration is highest in colostrum (7.0 g/L) and declines to ~1.0 g/L in mature milk [5]. In contrast, the concentration of Lf in bovine milk is substantially lower than human milk at a level of 0.83 g/L in colostrum and 0.09 g/L in mature milk [6].

Lf is an 80-kDa glycoprotein consisting of 703 amino acids and multiple sialic acid (Sia) residues attached to N-linked glycan chains [7, 8]. As a member of the transferrin family of iron-binding proteins, Lf shares more than 60 % homology at the amino acid level with transferrin [9, 10] and 68 % homology with human and bovine species. An important functional property of Lf is its high affinity for binding iron [7]. Lf also has multiple biological functions, including its ability to modulate immune function and its antimicrobial activity against viruses, bacteria, and fungi [11, 12]. It functions to facilitate iron metabolism, to promote bone growth, and to inhibit the growth of some human cancers [13–15]. In human clinical studies, Lf was shown to play a protective role in reducing the incidence of invasive fungal infections [16] and late-onset sepsis [17]. In very low birth weight neonates, Lf can prevent the development of necrotizing enterocolitis [5]. Thus, Lf, as a bioactive molecule, plays a critical role in boosting the immune system to modulate inflammation [13].

Lf can cross the blood-brain barrier via receptor-mediated transcytosis [18] and has suppressive effects on psychological distress [19]. These findings suggested a potential involvement of Lf in neural functions. The first 2 years of a child’s life is a critical age for early postnatal brain development [1, 2]. During this period, a series of important developmental events occurs in a highly sequential order. These include neuronal cell proliferation, differentiation, migration, and synaptic connections. These neural processes are of critical importance for the development of cognitive functions and the mental activity to acquire new knowledge to be integrated into responses, including perception, learning, memory, judgment, and problem-solving ability.

In order to test the hypothesis that milk Lf may have a beneficial impact on neurodevelopment, cognition, and memory, we initiated studies to investigate the molecular and cellular mechanisms underlying how Lf influences the developmental expression of genes and pathways. These analyses included global gene expression profiles, quantitative PCR (qPCR) validation, brain-derived neurotrophin factor (BDNF) protein, and polysialic acid (polySia) expression levels in selected regions of the hippocampus and frontal cortex in the brains of postnatal piglets.

Piglets have been chosen as our animal model because their brain structure and function closely resembles that of preterm human infants [20, 21]. The newborn piglet, similar to humans, is less developed and its body weight is relatively small in relation to its mature weight. Moreover, the piglet’s digestive system shares similar physiology and anatomical structure with human infants and has comparable nutrient requirements. This makes the piglet an ideally suited animal for the translational molecular neurobiology study of human infants [22, 23].

## Materials and Methods

### Animals

Three-day-old male domestic piglets (*Sus scrofa*, Landrace × Large White cross) were purchased from a commercial piggery in Xiamen City, China and randomly placed in the control (*n* = 16) and Lf treatment (*n* = 17) groups, based on the weight and litter information. All participating personnel were blinded as to the dose level of Lf that each piglet received throughout the study. The Animal Ethics Committee, Xiamen University, approved the study protocol.

### Animal Feeding

Piglets were fed a standard sow milk replacer diet containing a protein mixture consisting of soy/whey/casein (50:38:12) from 3 to 38 days of age, which is equivalent to about 10-month-old human infants [21]. Bovine milk Lf (DMV International, The Netherlands) was blended into the piglet’s milk replacer (Feed & Grow International Co. Ltd., China) at specified concentrations. The pig milk replacers were formulated such that the total protein intake remained the same between two groups, irrespective of the amount of added Lf. The amount of Lf in the final milk varied according to groups: 0.06 g/L (control, *n* = 16) and 0.6 g/L (Lf treatment, *n* = 17). These concentrations represented an approximate intake of 15 and 155 mg/kg/body weight/day, respectively. This dose was chosen because it is a level ~10-fold higher than Lf in the control milk and only ~8 % of the highest safe dose (2,000 mg/kg/day) recommended by the European Food Safety Agency (http://www.efsa.europa.eu/en/efsajournal/pub/2691.htm). We also analyzed the concentration of Lf, Sia, and iron in the experimental diets (Supplementary Table S1), confirming that the nutritional composition of the two diets was similar, except for the level of Lf. The procedure for feeding and care of piglets during the trial was carried out based on our previously published methods [22]. Body weight of the piglets was measured each morning before feeding using a digital scale (PRIS-Scale model: XK 3116, Chengdu Pris Electronic Co. Ltd, China). Milk intake and health status of the piglets were monitored and recorded daily.

### Sample Collection

On day 39 of age, piglets were euthanized by injecting pentobarbital (100 mg/kg) into the jugular vein. Approximately ~100 mg of tissues were immediately isolated from the hippocampus and prefrontal cortex, sliced, and incubated in 1.5 mL RNA Safer (R0424-02, Omega Bio-Tek, USA) in a RNase-free tube. The remaining segments of the brain were immediately packaged in prelabeled aluminum foil, frozen in liquid nitrogen, and stored at −80 °C for later analyses. Samples set into RNA Safer were kept at 4 °C overnight and transferred to −80 °C for subsequent analyses.

### Gene Microarray Analysis

Hippocampal tissues (~10–20 mg wet weight) were homogenized in a lysis buffer (Agencourt) purchased from Beckman Coulter, USA. Total RNA was prepared from each tissue sample using the Agencourt RNAdvance™ Tissue Kit (Beckman Coulter, USA) following the automated procedure described by the MICROLAB® STAR Liquid Handling Workstation (Hamilton Robotics GmbH Bonaduz GR, Switzerland) [24]. The quality of the RNA was monitored using an Agilent 2100 Bioanalyzer. Briefly, 250 ng of total RNA (integrity numbers >7.5) was used to generate double-stranded complementary DNA (cDNA), followed by in vitro transcription and complementary RNA (cRNA) labeling with biotin. Biotin-labeled cRNA (12.5 μg) was added to the hybridization mixture containing the control oligonucleotides, hybridization buffer, and water. A total of 18 hybridization mixtures, nine each for the Lf and control groups, were applied to a specialized Porcine Microarray Chip (Catalog No. 900625 GeneChip® Porcine Genome Array, Affymetrix). After hybridization for 16 h at 55 °C, the microarray chips were washed to remove nonhybridized material, stained with streptavidin, and then scanned with a GeneChip Scanner 3000 7G 4C w/7G Workstation. Signal intensities were determined and analyzed using the Affymetrix GeneChip Command Console software. All genes were imported into Partek Genomics Suite 6.5 software (Partek Inc., USA, www.partek.com) for one-way ANOVA analysis. Data were expressed as absolute intensities. Quality control of the data was carried out on all samples using a Pearson correlation matrix. No outliers were identified using the *principal component analysis* method with Partek software. After normalization, results were analyzed via log2, transformed, and analyzed by Student’s *t* test. Genes showing significant differences (*p* < 0.05) were selected and analyzed using the Ingenuity® System (Redwood City, CA, USA, http://www.ingenuity.com) and the Database for Annotation, Visualization and Integrated Discovery (DAVID, http://david.abcc.ncifcrf.gov/).

### Quantitative PCR Analyses

Total RNA from pig hippocampus (10 mg) was prepared and reversed transcribed into cDNA following instruction in the RevertAid First Strand cDNA Synthesis Kit (Fermentas, K1266, Thermo Fisher Scientific, USA). Twenty nanograms of cDNA were used for quantitative PCR in a total volume of 10 μL in 384-well plates. The relative expression value for each gene was calculated as gene quantity divided by a normalization factor (NF), which was derived from GeNorm Software (http://medgen.ugent.be/~jvdesomp/genorm/) using glyceraldehyde 3-phosphate dehydrogenase (GAPDH) and HPRT1 as the reference genes. Gene quantity was determined according to a relative standard curve, which was prepared using serial dilution of pooled total cDNA at concentrations of 80, 40, 20, 10, 5, 2.5, 1.25, and 0.625 ng/μL. All real-time PCR reactions were set up with the use of the MICROLAB® STAR Liquid Handling Workstations (Hamilton Robotics GmbH Bonaduz GR, Switzerland) [24] and analyzed in triplicate. qPCR primer sequences are described in Supplementary Table S2. Quantification was carried out using the ABI Prism 7900 HT Sequence Detector (Applied Biosystems, USA). The mean value of the control group was used as the control calibrator, and the Lf group was expressed as *n*-fold ratio in graphs, compared with the calibrator.

### ELISA Analyses

Briefly, ~100 mg of hippocampal tissue was homogenized in 1 mL of cold HENT lysis buffer (50 mM 4-(2-hydroxyethyl)-1-piperazineethanesulfonic acid (HEPES) (pH 7.5), 2 mM EDTA, 50 mM NaCl, 2 % Triton X-100, 1 mM dithiothreitol (DTT), 1 mM phenylmethylsulfonyl fluoride (PMSF)) in a prechilled glass homogenizer using 15 vertical strokes. The homogenates were transferred to a 2-mL Eppendorf tube and placed on ice for 1 h before centrifugation at 20,000×*g* at 4 °C for 20 min. Protein levels in the supernatant were quantitatively determined using the BCA Protein Assay Kit (23227, Thermo Scientific, USA). BDNF levels in the piglet hippocampus were determined using a sandwiched ELISA kit (E-EL-P0184, Elabscience, China). The absolute value of BDNF in each sample was determined by dividing the amount of BDNF by the total protein concentration. Results are expressed as picogram BDNF per milligram protein.

### Immunofluorescence

Consecutive coronal sections of 20 μm thickness from the piglet’s hippocampus were prepared using a freezing microtome (LEICA CM 1950, Germany). Brain sections were fixed in ice-cold 4 % paraformaldehyde for 15 min, rinsed in phosphate-buffered saline (PBS), and permeabilized with 0.3 % Triton X-100 for 30 min, followed by blocking with 0.3 % goat serum in PBS (room temperature). The thin sections were incubated overnight (4 °C) with a primary antibody specific for detecting the polySia moiety of polySia-neural cell adhesion molecule (NCAM) (1:200; MAB5324, Millipore, USA). After washing with PBS, sections were incubated with Alexa Fluor 488-conjugated secondary antibody (1:200; 115-545-075, Jackson ImmunoResearch, USA) for 1 h at room temperature. All sections were counterstained with 4′,6′-diamidino-2-phenylindole (DAPI) (H-1200, Vector Laboratories, USA). Images were obtained and analyzed using a confocal microscope (FV1000, Olympus, Japan).

### Western Blotting Procedure

The amount of total protein present in the hippocampus and prefrontal cortex tissues was quantitatively determined using the BCA Protein Assay Kit. To determine the expression level of polySia-NCAM in the hippocampus, ~100 mg of tissue was homogenized by mechanical disruption in cold lysis buffer containing 50 mM HEPES (pH 7.5), 2 mM EDTA, 50 mM NaCl, 2 % Triton X-100, 1 mM DTT, and 1 mM PMSF, as a protease inhibitor. For Western blot analyses, 20 μg of protein from each hippocampal sample was subjected to sodium dodecyl sulfate polyacrylamide gel electrophoresis (SDS-PAGE) (5 % stacking and 7 % running gel). Electrotransfer to the polyvinylidene difluoride (PVDF) membrane was carried out at 70 V for 3 h. For analysis of the expression levels of cyclic adenosine monophosphate (cAMP) response element-binding protein (CREB) and phosphorylated CREB, ~50 mg of hippocampal protein was homogenized in 500 μL of cold HENT lysis buffer with phosphatase inhibitors (04906845001, Roche, Switzerland) using a high-speed disperser and treated with ultrasonication for several seconds before centrifugation at 20,000×*g*/10 min at 4 °C. Protein levels in the supernatant were quantified using the BCA Protein Assay Kit. Eighty micrograms of protein for each sample was subjected to SDS-PAGE (5 % stacking and 10 % running gel) and then electrotransferred to a PVDF membrane at 80 V for 1.5 h. The PVDF membranes were blocked with 5 % skim milk for 1 h and then incubated with primary antibodies at 4 °C overnight. The concentrations of antibodies for detecting polySia-NCAM (MAB5324, Millipore) and phosphorylated CREB (9191S, Cell Signaling Technology, USA) or CREB (9197S, Cell Signaling Technology, USA) were 1:1,000 and 1:500, respectively. GAPDH was used as a control for the amount of protein loaded on each gel and detected using an anti-GAPDH mAb (MAB5718, R&D Systems, USA) at 0.05 μg/mL. After washing the membrane three times with TBST (10 mM Tris, 150 mM NaCl, 0.05 % Tween-20), the membranes were incubated with secondary antibody (goat-anti-mouse IgG, BA1050, BOSTER, China; goat-anti-rabbit IgG, 7074, Cell Signaling Technology, USA) for 1 h at room temperature. After a second cycle of washing with TBST, the immune complexes were detected by enhanced chemiluminescence (CW0049A, CWBIO, China; RPN2235, GE Healthcare, USA). Membranes were then exposed to an X-ray film (XBT-1, Kodak, USA). Quantification of the protein bands was carried out by scanning the films using the Image Analyses Software (Quantity One, Bio-Rad, USA). The density of bands on all films was determined under nonsaturating conditions.

### Assessment of Learning Capability

Our published method, developed using an eight-arm radial maze, was used to evaluate the piglets’ learning and memory capability [22]. The formal learning tests began on postnatal day 23. In this radial maze assay, two tests were carried out: an “easy” learning task and a “difficult” learning task, as described in Supplementary Fig. S1. A total of 40 trials in both the easy and difficult learning tasks were carried out [22]. The criteria to learn the visual cue was a maximum of 1 mistake in 3 consecutive trials, a method validated earlier for piglets [22].

### Statistical Analyses

Differences in learning, defined as the number of trials required to learn the visual cue, were compared using the Kaplan-Meier survival analysis with Cox regression to examine potential covariates that may influence learning [22]. Comparison of messenger RNA (mRNA) levels for BDNF; protein expression levels for BDNF, CREB, pCREB, and polySia-NCAM; body weight gain; and the level of plasma stress hormones between the Lf and control groups were carried out using Student’s *t* test. Data are expressed as mean ± SEM, and a significance level of 0.05 was used. All statistical analyses were completed with the use of SPSS for Windows 19.0 (SPSS, Inc., Chicago, IL, USA).

## Results

### Global Gene Transcription Profiling in the Hippocampus of Postnatal Piglets

**Fig. 1:**
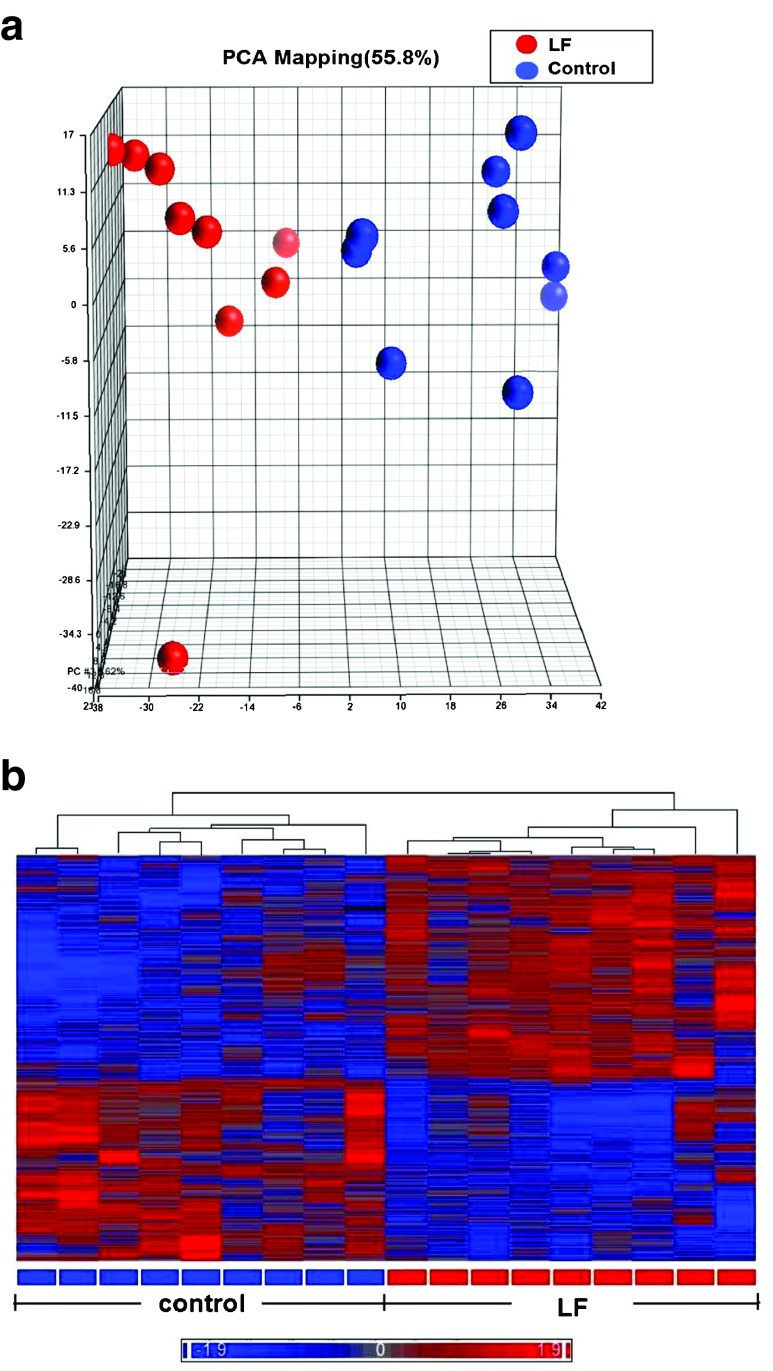
Gene profiling microarray analysis of hippocampal tissues from postnatal piglets after Lf intervention: **a** “principal component analysis” (PCA) mapping between Lf treatment (*red*, *n* = 9) and control group (*blue*, *n* = 9) using Partek Genomics Suite 6.5 software (www.partek.com). *Each symbol* represents a single sample. **b** A heat map representative of the 1,187 genes that were differentially expressed between the control and Lf groups (filter criteria: fold change 1.1 and *p* < 0.05). *Blue*: genes that were downregulated, *red*: genes that were upregulated. The normalized expression value is indicated at the bottom of the figure

We sought to determine how Lf intervention might impact gene expression profiling on the genomic scale in the brains of postnatal piglets. Our primary interest was in the hippocampus, an area of critical importance for cognition and learning. To achieve this aim, we used a porcine Affymetrix GeneChips containing 23,937 probe sets to interrogate 23,256 transcripts in pig, which represents 20,201 *S. scrofa* genes to map the gene expression profile in the hippocampus of piglets receiving Lf and a control group whose diet was not supplemented with Lf. For both groups, there was no difference in the quality of RNA, hybrid mixture profiles, or the signal intensity in the Pearson correlation map. A total of 1,187 genes were differentially expressed between the control and Lf groups, based on our filter criteria (fold change 1.1 and *p* < 0.05). The Partek Genomics Suite 6.5 software (Partek Inc., www.partek.com) was used for principal component analysis (PCA) to determine the key variables from complex data sets. These analyses showed that Lf was the major effect factor (Fig. 1a). A hierarchical clustering and heat map were also generated, which showed the differential transcriptomes between the control and Lf groups (Fig. 1b). Blue represents those genes that were downregulated, while red represents genes that were upregulated for the normalized expression value in the color-coded scale, as shown in the bottom of the figure. The 1,187 genes were further subjected to pathway analyses using the Ingenuity Pathway Analysis (IPA) software (Ingenuity® Systems, http://www.ingenuity.com). Importantly, as shown in Table 1, a global beneficial effect of Lf on neurodevelopment and cognitive function was observed, as evidenced by the modulation of a wide range of neuronal processes including an increase in cellular protrusions, microtubule dynamics, formation and organization of neurite outgrowth, cytoskeleton formation, and a decrease in anxiety.

**Table 1:** Summary of molecular and cellular functions related to the nervous system affected by dietary lactoferrin

Molecular and cellular functions in the nervous system	*p* value	Predicted activation state	Regulation *z*-score	Number of molecules Involved
Formation of cellular protrusion	1.70E-07	Increase	2.294	39
Microtubule dynamics	2.70E-07	Increase	2.504	49
Organization of cytoplasm	1.09E-06	Increase	2.319	57
Formation of cytoplasm membrane projections	5.39E-06	Increase	2.305	28
Organization of cytoskeleton	5.73E-06	Increase	2.505	51
Outgrowth of neurites	0.00779	Increase	2.505	16
Formation of neurites	0.00824	Increase	2.076	9
Anxiety	0.00575	Decrease	−2.717	10

Statistical significance of each function was calculated via a right-tailed Fisher’s exact test in the IPA software (Ingenuity Systems, USA). The predicted activation state is determined based on the value of regulation *z*-score (>2: increase; <−2: decrease)

### Lf Upregulates the BDNF Neurotrophic Signaling Pathway

**Fig. 2:**
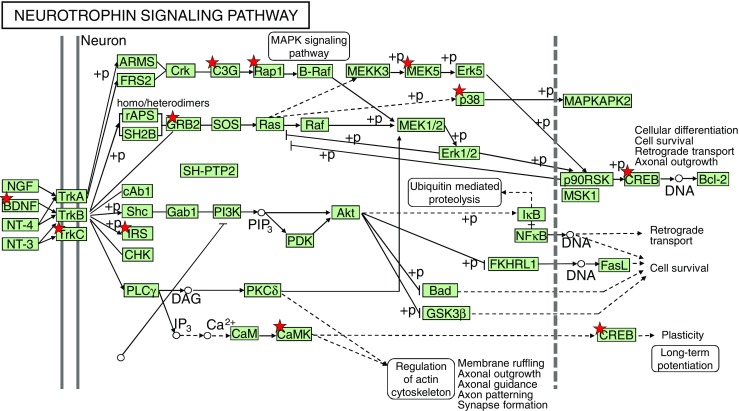
Lf intervention upregulates the neurotrophin-signaling pathway in postnatal piglets. The activated genes are denoted with a *red star*: *p* < 0.05~0.001. Genes increased by Lf intervention vs. the control group of piglets are summarized in Table 2. A number of functional studies have shown that this signaling cascade network can affect neural development, neuronal structure construction, and long-term potentiation, as described in the text. The Lf-induced increased in BDNF expression was confirmed by qPCR, as shown in Fig. 3

BDNF is one of a family of crucial neurotrophic growth factors that are expressed at high levels in the hippocampus and cortex. BDNF has several important functional roles in neuronal transmission and plasticity and participates in the formation of memory and learning, the survival of neurons, and in promoting growth and differentiation of new neurons and synapses [25]. Our Affymetrix gene microarray analysis showed that the BDNF signaling pathway was activated by Lf supplementation. As shown in Table 2, the expression level of mRNA encoding for BDNF was upregulated 1.3-fold in the hippocampus. Concomitant with the increased expression of BDNF was an upregulation in the expression level of other key genes in the BDNF signaling pathway, including Trk3, IRS1, GRB2, CAMK1, MAPK, SP1, and CREB1 (*p* < 0.01, Fig. 2, red star). Upregulation of specific genes in response to Lf in the BDNF signaling pathway is summarized in Table 2. Of particularly note is the upregulation of the affinity binding of BDNF to BDNF receptors and their subsequent dimerization within the membrane, which stimulates several intracellular signaling cascades [26]. These cascades include the mitogen-activated protein kinase/extracellular signal-regulated protein kinase (MAPK/ERK), phospholipase Cg (PLCg), and the phosphoinositide 3-kinase (PI3K) pathways [27–29]. Activation of these pathways results in phosphorylation of multiple transcription factors, including the CREB, which then initiates the de novo expression of different target genes.

**Table 2:** Summary of genes involving the BDNF neurotrophin-signaling pathway that were up- or downregulated by dietary lactoferrin supplementation

Gene name	Probe set ID	*p* value	Fold change (compared with the control group)
BDNF	Ssc.16243.1.S1_at	0.004338	1.30597
IRS1	Ssc.7304.2.A1_at	0.005528	1.19099
Trk3	Ssc.4915.1.A1_at	0.000455	1.24593
PI3K	Ssc.11109.1.S1_at	0.007745	−1.17788
RAP1A	Ssc.24315.1.S1_at	0.002479	−1.16274
CAMK	Ssc.2491.1.S1_at	0.009374	1.18927
MAPK11	Ssc.29722.1.S1_at	0.034919	1.1143
MAPK12	Ssc.6498.1.A1_at	0.002471	1.10944
CREB1	Ssc.8827.1.S1_at	0.006465	1.2
SP1	Ssc.18559.1.S1_at	0.000802	1.1819
MYC	SscAffx.8.1.S1_at	0.035227	1.16126
ESR1	gi:52346219_at	0.044021	1.17212

**Fig. 3:**
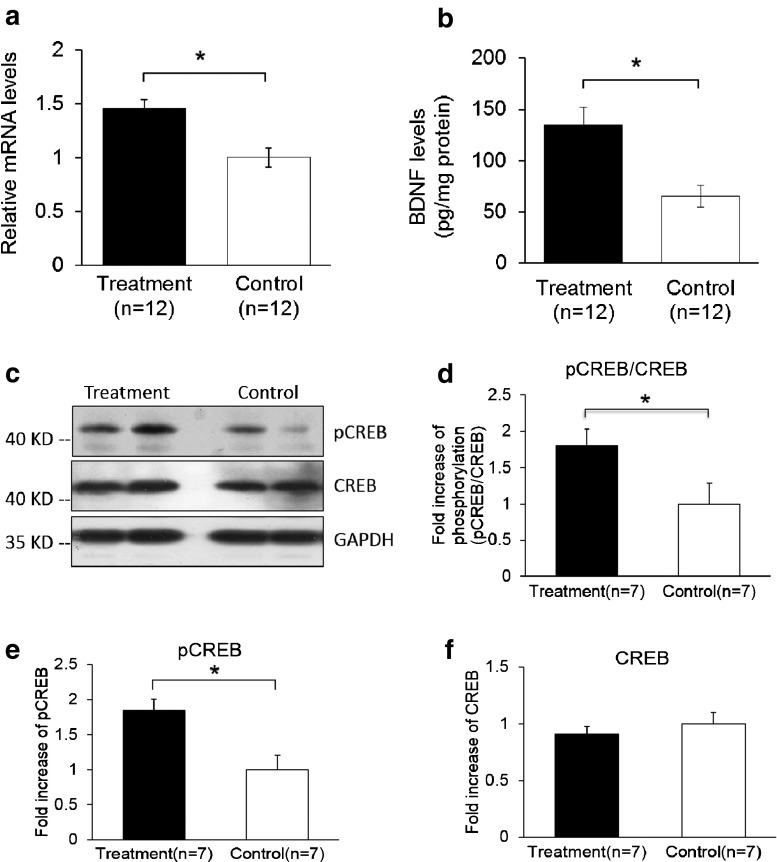
Lf upregulates BDNF gene expression levels and downstream signal transduction in the hippocampus of postnatal piglets: **a** mRNA transcriptional level of BDNF in the Lf-treated group (qPCR method); **b** protein abundance levels encoding for BDNF in the Lf-treated group (ELISA method); **c** phosphorylation of the transcriptional factor, CREB, was analyzed by Western blotting using antibodies specific for recognizing phosphorylated CREB, total CREB, and GAPDH, respectively; **d**–**f** quantitative Western blot analysis of **d** phosphorylation of CREB, **e** phosphorylated CREB, and **f** total CREB expression in the Lf-treated group (*n* = 7) vs. the control group (*n* = 7). Data is mean ± SEM. *p < 0.05

A quantitative PCR analysis was then carried out to validate the gene microarray findings, which confirmed that the level of BDNF was elevated by Lf. For example, the mRNA level for BDNF in the Lf-treated group was 1.5-fold higher than the control group (*p* = 0.001, Fig. 3a). Importantly, these analyses also showed that the level of the BDNF protein in the hippocampus was increased 2.1-fold by Lf (*p* = 0.024, Fig. 3b). It is well established that activation of the BDNF signaling pathway leads to enhanced phosphorylation and nuclear translocation of CREB [29]. The phosphorylation of CREB at serine 133 (pCREB) induces gene transcription and plays a major role in initiating learning and memory processes [30]. To examine the functional consequences of the Lf-induced message levels for CREB phosphorylation, we determined the total protein levels of CREB and pCREB. Our findings show that Lf upregulated the steady-state level of phosphorylated CREB in the hippocampus of piglets (*p* < 0.05, Fig. 3c, d), and the results did not change when pCREB expression between the two groups was analyzed separately (*p* < 0.05, Fig. 3e). There was, however, no significant difference in total CREB protein expression between the two groups (*p* > 0.05, Fig. 3f). On the basis of these new findings, we postulate that the Lf-induced increase in BDNF levels, and BDNF’s subsequent effect on the signal transduction cascade, may be the underlying molecular mechanism to explain how Lf enhances cognition and memory, given the seminal role that BDNF plays in such a myriad of neural functions.

### Neuroplasticity in the Hippocampus

**Fig. 4:**
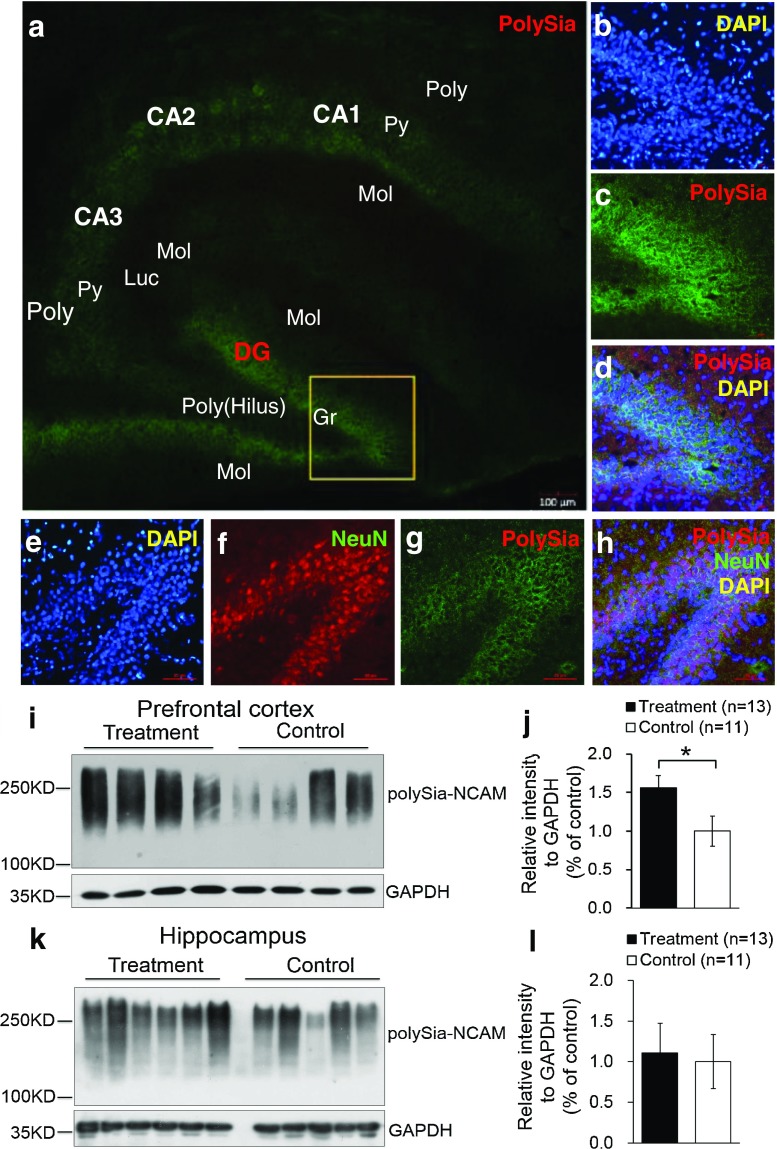
Lf upregulated polySia-NCAM expression in selected regions of the brain in postnatal piglets: **a**–**d** immunofluorescent staining of polySia-NCAM in the hippocampus (*green*, polySia-NCAM; *blue*, DAPI (**a**). Magnification (×4) images of polySia-NCAM staining in the hippocampus; *DG* dentate gyrus; *CA1*, *CA2*, and *CA3*: Cornu ammonis 1, 2, and 3; *Poly* stratum (*S*) polymorphum; *Py* S. pyramidale; *Mol* S. moleculare; *luc* S. lucidum as one part of the Mol in CA3; *Gr* granular cell layer and the S polymorphum in DG, also called the hilus of DG. **b**–**d** Higher magnification (×20) image of DAPI (**b**), polySia-NCAM (**c**) and **d** the merged images of **b** and **c. e**–**h** Immunofluorescent staining of the dentate gyrus in the hippocampus with DAPI (*blue*, **e**); NeuN (*red*, **f**); polySia-NCAM (*green*, **g**); and the merged images of polySia-NCAM, NeuN, and DAPI (**h**). Lf intervention increased the level of polySia-NCAM expression in the hippocampus and prefrontal cortex of postnatal piglets as confirmed by Western blot analyses. GAPDH served as the control for protein loading. **i** Levels of polySia-NCAM in the prefrontal cortex of four Lf-treated piglets vs. the control group (*p < 0.05, Student’s *t* test). **j** Quantitation of the Western blot analysis of the Lf-treated piglets (*n* = 15) vs. the control group (*n* = 11); **k** Western blot analyses of polySia-NCAM expression in the hippocampus of the Lf-treated piglets vs. the control group (*p* > 0.05, Student’s *t* test); and **l** quantitation of the Western blot analysis of the Lf-treated piglets (*n* = 13) vs. the control group (*n* = 11; (*p* > 0.05)

The polySia moiety of NCAMs is a key glycan known to mediate a number of brain functions critical for normal neural development, postnatal growth, and survival [2, 31]. In 2008, Kanato et al. made the unexpected discovery that polySia chains with a degree of polymerization of at least 12 Sia residues bound directly to the BDNF dimer under physiological conditions to form a large Mr complex of >2,000 kDa [32]. Importantly, the BDNF-polySia complex can bind the BDNF receptors to upregulate growth and survival of neuroblastoma cells [32]. Large polySia complexes can also be formed with other neurotrophic factors including nerve growth factor, neurotrophin-3, neurotrophin-4, and FGF2. This important new finding has led to an entirely new function for polySia as a “reservoir” for bioactive, neurotrophic molecules and growth factors [33]. In our study, a relatively higher level of polySia-NCAM was expressed in the hippocampus (Fig. 4k, l) and prefrontal cortex (Fig. 4i, j) of piglets whose diet was supplemented with Lf, compared with the control group (*p <* 0.05, Fig. 4i–l). Immunofluorescent staining showed that high levels of polySia-NCAM were expressed in subregions of the dentate gyrus (DG) and the Cornu ammonis (CA)1, CA2, and CA3 within the hippocampus (Fig. 4a). These are regions of the brain where neurogenesis and processes for higher critical neural functions take place, including learning, memory, and spatial coding. Higher magnification (×20) images of DAPI, polySia-NCAM, and the merged immunofluorescent staining of DG subregions showed high levels of polySia expression that correlated with the large number of neuronal cells (Fig. 4b–d). Furthermore, the merged immunofluorescent staining of DAPI, polySia-NCAM, and neuronal nuclei (NeuN), the latter a marker of mature neurons, showed that polySia was heavily expressed in the neuronal cell bodies (Fig. 4e–h). These findings suggest that the sialyl moieties and/or the peptide moiety of Lf may be a key molecular player in increasing neuroplasticity and facilitating long-term memory (LTM) consolidation during maze learning in developing piglets. To our knowledge, these new findings have not been previously reported.

Also of potential interest is the variation in the levels of polySia-NCAM expressed in the hippocampus and prefrontal cortex of four individual outbred piglets (Fig. 4i, k). Some piglets fed Lf, for example, expressed 2-fold higher levels of polySia-NCAM compared with the control group. This is most evident in the prefrontal cortex (Fig. 4i, j) but is also seen in the hippocampus (Fig. 4k, l). The reason for this variation is not known. Our findings of elevated levels of polySia-NCAM in the hippocampus and prefrontal cortex likely represent the lower level of this glycan that is actually upregulated by Lf because it is difficult to quantify by standard SDS-PAGE electrophoresis methods such high molecular mass complexes that cannot enter the polyacrylamide running gel [32].

### Assessment of Learning Performance

**Fig. 5:**
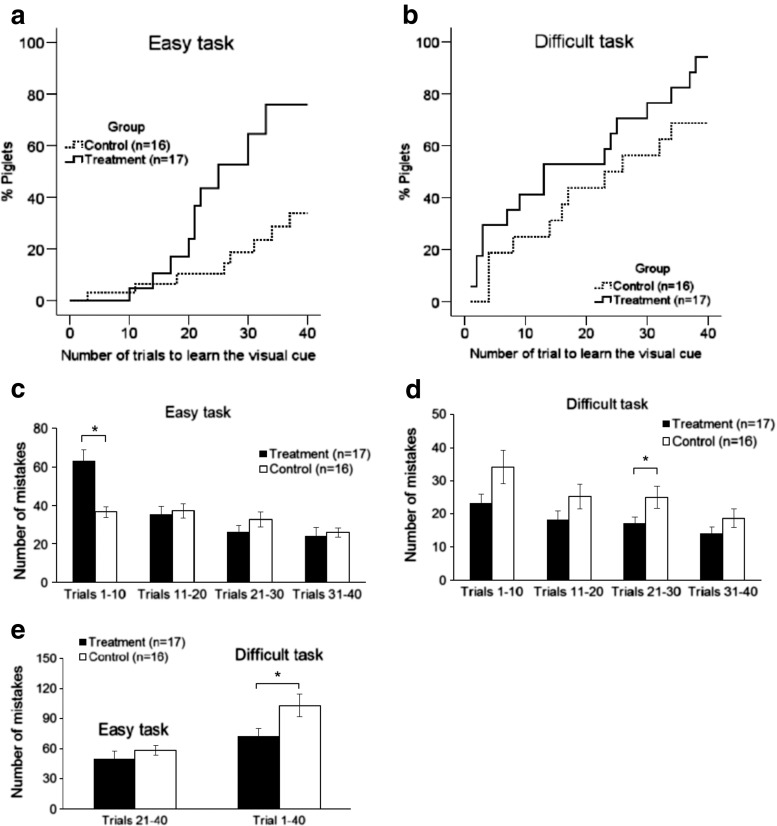
Assessment of the piglets’ learning and memory capacity. **a**–**e** Assessment of learning capacity: **a** easy learning task: Kaplan-Meier survival analysis with Cox regression, and generalized linear model log-rank test with total number of mistakes in the first 20 trials as a covariance of analysis (*p* = 0.001). **b** Difficulty learning task: Kaplan-Meier survival analysis with Cox regression and generalized linear model log-rank test with total number of successes in last 20 trials as a covariance of analysis (*p* = 0.018). **c**, **d** Number of mistakes made during different stages of learning (per 10 trials) in the easy and difficult learning task, respectively. **e** Number of mistakes in the second half of the easy learning task (trials 21–40) and all 40 trials of the difficult learning task

The eight-arm radial maze was first designed by Olton and Samuelson in 1976 to measure spatial learning and memory in rats [34]. Since that time, the maze has been improved and is a consolidated paradigm for the evaluation of spatial learning, associative learning, and memory in many animal species, including piglets [22]. In both easy and difficult learning tasks, the Lf group learned the visual cue more rapidly (video record of Lf-treated “smart” piglet compared with the video record of the control or “dumb” piglet). In the easy learning task, for example, only 33.5 % of the control group of piglets reached the learning criteria within 40 trials, compared with 75.5 % in the Lf group (*p* = 0.001, Fig. 5a, Kaplan-Meier survival analysis with Cox regression and generalized linear model log-rank test), when the total number of mistakes in the first 20 trials was used as a covariance. This is because learning in the first 20 trials of the easy task is predominantly by “trial and error” [22]. In the difficult learning task, however, 96.8 % of the piglets in the Lf group reached the criteria within 40 trials, compared with only 78.7 % in the control group (*p* = 0.018, Fig. 5b, Kaplan-Meier survival analysis with Cox regression and generalized linear model log-rank test), when the total success in the last 20 trials (reinforced learning) was considered as a covariance. More piglets reached the learning criteria in the difficult task than in the easy task because learning easier tasks guides the learning of more difficult tasks, since learning is more effective as the tasks become more difficult [22, 35]. Furthermore, in the easy learning task, the piglets that learned more rapidly underwent continuing reinforcement that carried over to the more difficult learning task. These findings remained unchanged when adjusted for body weight, rate of weight gain, or the level of stress hormones in the blood (Supplementary Fig. S2).

Since learning is the acquisition of new knowledge or skills through experience or practice, we analyzed separately the results of each 10 trials. On the first day of the easy learning task, the Lf group made more mistakes than the control group, but the Lf group showed a significant improvement in learning, as the total number of mistakes was less than the control group in the last three sets of the 10 trials (Fig. 5c). In the difficult learning task, the Lf group made fewer mistakes than the control group when each of the 10 trials was analyzed separately, or when all mistakes were analyzed in the total of 40 trials (*p <* 0.05, Fig. 5d, e). These results further support our conclusion that Lf at 155 mg/kg/day facilitated cognition and learning in postnatal piglets.

In the present study, piglets that reached the learning criteria in both the easy and difficult tasks continued to learn throughout the 40-trial test period. This aspect of “reinforcement learning” was expected and unavoidable because if learning had been stopped when the piglets reached the criteria early, then the elapsed time between reaching this criteria and the memory test would be different among different piglets [22]. Alternatively, if the memory test was administered immediately after the piglets reached the criteria, then the developmental age of piglets would have been different at the time of the memory test and before euthanasia [30].

### Body Weight and Plasma Cortisol Levels

The mean (±SEM) body weight of piglets at 3 days of age was the same in the control and Lf-treated groups (2.0 ± 0.08 kg). The piglets gained weight at similar rates from days 3 to 39 in the two groups (*p* > 0.05, Supplementary Fig. S2a). Thus, Lf did not influence the growth and development of postnatal piglets during the study period. Further, no significant differences were found between the two groups in the level of plasma cortisol and adrenocortical hormones throughout the study (*p* > 0.05, Supplementary Fig. S2b, c). This suggests that there was no significant difference in the stress levels between the two groups of piglets.

## Discussion

The interaction between cognitive development and nutrition is an extremely complex process involving multiorgan physiology and molecular and cellular mechanisms regulating expression levels of genes, proteins, and metabolites. In this study, we have tested the hypothesis that Lf supplementation may enhance neurodevelopment and cognition. Accordingly, we used a global gene microarray method to investigate the gene and protein expression levels in the hippocampus of postnatal piglets whose diet was supplemented with Lf. It was unanticipated to discover that Lf changed the expression profiles of such a large number of genes involved in many different neuronal signaling pathways, including those associated with neurite outgrowth, protrusion and cytoskeleton formation, and decreased anxiety. These gene microarray analyses support our findings of a positive effect of Lf on neuronal and cognitive functions. Importantly, we found that Lf elevated the transcriptional and posttranslational levels of BDNF in the hippocampus and its signaling transduction pathway. Given the significance of the neurotrophin family of nerve growth factors, particularly BDNF in neuronal development and plasticity of nerve cells, our findings show that Lf functions by increasing the level of BDNF, with the subsequent upregulation of the expression level of phosphorylation of CREB. Considerable evidence supports the role of BDNF as a key regulator for long-term potentiation (LTP) and suggests that BDNF may serve as a “plasticity-related protein” [36]. BDNF has pleiotropic effects on neuronal development and synaptic plasticity, which is the foundation of circuit formation and cognitive function. BDNF’s action is regulated at multiple molecular levels, including transcription, mRNA targeting, BDNF protein processing, and intracellular trafficking [37]. Although the signaling pathways activated by the interaction of BDNF during learning and memory are not well understood, different signaling cascades mediated by tropomyosin-related kinase B (TrkB) receptors, which are known to be coupled to the activation of the Ras/ERK, PI3K/Akt, and phospholipase C-γ (PLC-γ) pathways, have been reported [27]. Concomitant with the Lf-induced upregulation of BDNF was our gene microarray results showing that several transcription factors were also upregulated in the BDNF signaling pathway, including MYC, RAPGEF1, and estrogen receptor (*p <* 0.05, Table 2). The high expression level of mRNA for SP1, for example, involves many cellular processes, including cell differentiation, cell growth, apoptosis, immune responses, response to DNA damage, and chromatin remodeling [38]. The mRNA levels of GRB2, a gene related to developmental processes including cell proliferation in various organisms [39], and MAPK, a gene involved in regulating diverse cellular programs by relaying extracellular signals to intracellular targets [40], were also upregulated by Lf. MAPK/ERK plays a critical role in protein synthesis-dependent neural plasticity. The CAMK gene has the hallmark features regulating a form of molecular memory that is important in LTP of excitatory synapse strength and memory formation [41]. Major genes that are modulated by Lf are shown in Fig. 2 and summarized in Table 2. Although the filter criteria in our gene expression study was chosen as >1.1-fold change and *p* < 0.05 between the Lf and control groups, the RNA preparation and microarray analyses were carried out using the MICROLAB® STAR Liquid Handling Workstation (Hamilton Robotics GmbH Bonaduz GR, Switzerland). This analytical automatic sampling method was used because of its greater reliability for technical interexperiment variation compared with manual analyses [24]. Further, high-quality RNA samples (RNA integrity number >8) were used and quality control for all sample analyses was utilized throughout our microarray analysis, as described under “Materials and Methods” section. In addition, we also carried out a TaqMan® Gene Expression Assay based on real-time PCR and used these data as the reference to evaluate the performance of the microarray platforms between Lf and control piglets. We found that the TaqMan® gene expression assay data corresponded well with the microarray data. For example, the fold change in BDNF gene assay results was 1.3-fold in the Lf-supplemented piglets, compared with the control piglets, in the microarray assay and 1.5-fold in the qPCR (TaqMan®) experiments. Thus, these results confirmed that the effect of Lf on regulating these signaling pathway genes is both significant and reliable.

Interestingly, our gene microarray analyses further showed that the level of mRNA encoding for PI3K was downregulated (Fig. 2; Table 2). The pathway from GRB2 and IRS to PI3K, however, is not a single reaction but rather requires multiple steps in the signaling pathway [25, 27, 28]. Thus, each of these identified genes does not act in a linear sequence but rather in a sequential and complex network [42]. These signaling cascades are used in many different contexts and by other growth factors and cytokines [29]. Further studies on the role of Lf in regulating PI3K in postnatal piglets will be required to more fully understand this aspect of the BDNF signaling pathway. Overall, an understanding of the exact molecular mechanism of how Lf increases the de novo mRNA levels for BDNF is presently unknown but is a problem beyond the scope of our present study and a focus of our future research.

CREB, a transcription factor downstream of BDNF/TrkB-activated signaling pathway, is widely recognized as a critical regulator of gene expression in the central nervous system [26] because it contributes to neuronal plasticity, LTP, memory, fear conditioning, circadian rhythm entrainment, and neuron survival [43, 44]. Previous studies provided evidence that CREB proteins were involved in the formation of LTM in neurons in the invertebrates *Aplysia* and *Drosophila melanogaster* and in vertebrates including mice and rats [45, 44]. The role of CREB in memory is considered a major pathway leading to consolidation of synaptic plasticity and also a parallel pathway modulating intrinsic excitability [45]. We have shown that the level of phosphorylated CREB, an important nuclear target of PKA involved in memory processes, was significantly increased by Lf compared with the control group (Fig. 3c, d). This finding, along with the results showing Lf increased polySia-NCAM expression in the hippocampus and prefrontal cortex (Fig. 4i–l), thus provides a potential molecular basis for understanding the beneficial role of Lf in postnatal neural development and cognition, chiefly in learning and memory. Our quantitative analysis confirmed that Lf is not a major carrier of Sia in milk, but that its sialyl moiety, similar to the sialyl moieties on glycomacropeptide, may be critical for early neurodevelopment and cognition in newborns [2, 22].

PolySia plays a critical role in neural development by modulating the adhesive property of NCAM and is therefore implicated in a wide range of neurodevelopmental morphogenic events, including cell migration, neurite outgrowth, path-finding, sprouting, regeneration, and synaptic plasticity [46, 47]. Several studies have shown that learning increases the incorporation of Sia into the brain [22]. Cells within the hippocampal area, neurons in the entorhinal cortex, and the septal nuclei, for example, showed significant increases in polysialylation after training [48]. Mice deficient in polySia-NCAM showed a reduction in the size of the olfactory bulb and a defect in spatial learning and memory [49]. These findings suggest that it is the cellular behavior in response to task-associated stimuli, rather than retrieval from previously stored task-associated memory, that is responsible [50]. This leads to the postulate that transient polysialylation may be more important in processing information than storage of information [51]. Our study shows that Lf increased the expression level of polySia in both the hippocampus and prefrontal cortex, suggesting that transient changes in the extent of polysialylation may be a key event leading to altered synaptic plasticity required for increased learning, STM and LTM. Additionally, studies in rats showed that transient polysialylation played a key role in consolidating memory in the dentate gyrus [52].

Dramatic changes in neurodevelopment and a major transformation in cortical organization occur prenatally and during early postnatal development [53]. The advantageous characteristics of brain structure, physiology, and genome similarity in the brains of piglets compared with the brains of rodents have led to an increase in the use of postnatal piglets for neurodevelopment and cognitive studies. In the present study, we have shown that piglets whose diet was supplemented with Lf showed enhanced learning in both easy and difficult learning tasks (Fig. 5a, b). These findings are also consistent with those of Ahissar and Hochstein [35] who showed that easy learning tasks guide or facilitate the learning of more difficult tasks. In such learning studies, a critical aspect is the method used to assess learning capacity.

One of the more dramatic changes in neurodevelopment and a major transformation in cortical organization occurs prenatally and during early postnatal development [53]. Among mammals, piglets share a high similarity with human infants in the brain growth spurt, the gross anatomy, and growth pattern in which the brain undergoes rapid growth and maturation [54, 23]. Piglets are also cooperative animals and can rapidly learn classical and operant conditioning tasks [23]. Thus, the piglet is an ideal animal model to study the effect of nutritional intervention on neurodevelopment and cognitive function for translational studies that are of potential significance to human infants.

In summary, the present studies have established that Lf intervention improved cognition in postnatal piglets and that this acquisition correlated with changes in the expression of a variety of genes involved in neurodevelopment and cognition. These changes were mediated, at least in part, by the Lf-induced upregulation of the BDNF signaling pathway. This effect led to increased levels of BDNF and its downstream signaling transduction of the phosphorylation of CREB, a protein of known importance in neurodevelopment and cognition. The high expression level of polySia-NCAM in the hippocampus and prefrontal cortex also correlated with increased cognition and learning. Our findings provide new, fundamental insight into the molecular mechanisms underlying the role of Lf in neurodevelopment, learning, and memory capacity in postnatal piglets, findings that have not been previously reported. Our future studies will examine the detailed molecular mechanisms underlying how Lf activation of the BDNF signaling pathway is related to the upregulation of polySia-NCAM and the relationship of these two key pathways to enhance neurodevelopment and cognition.

## Electronic supplementary material

Below is the link to the electronic supplementary material.

ESM 1 (DOCX 198 kb)

ESM 2 (MPG 1816 kb)

ESM 3 (MPG 3432 kb)
