# Supplementary material for: Lactoferrin Promotes Early Neurodevelopment and Cognition in Postnatal Piglets by Upregulating the BDNF Signaling Pathway and Polysialylation
Source: Mol Neurobiol. 2014 Aug 23;52(1):256–69. doi: 10.1007/s12035-014-8856-9 (PMC4510916; doi:10.1007/s12035-014-8856-9)
Supplement: Supplementary file 1 — (DOCX 198 kb) [file 12035_2014_8856_MOESM1_ESM.docx]

**Supplementary material**

(Supplementary Fig. S1-S3, Table S1 and S2, Movie S1 and S2)

**Lactoferrin Promotes Early Neurodevelopment and Cognition in Postnatal Piglets by Up-regulating the BDNF Signaling Pathway and Polysialylation**

Yue Chen, Zhiqiang Zheng, Xi Zhu, Yujie Shi, Dandan Tian, Fengjuan Zhao, Ni Liu, Petra S Hüppi, Frederic A Troy II and Bing Wang

**Inventory of Supplementary**

**Fig. S1** Schematic diagram of 8-arm radial maze and the visual cues

**Fig. S2** Body weight gain and plasma hormone levels of ACTH and cortisol in the Lf and control groups during the study period

**Table S1** Concentration of lactoferrin, sialic acid and iron in the experimental diets

**Table S2** Primer sequences and accession number used in real-time PCR experiments for BDNF

**Movie S1** Video record of a smart piglet searching food in 8-arm radial maze

**Movie S2** Video record of a dumb piglets searching food in 8-arm radial maze

**Supplementary Figures**

**Fig. S1**


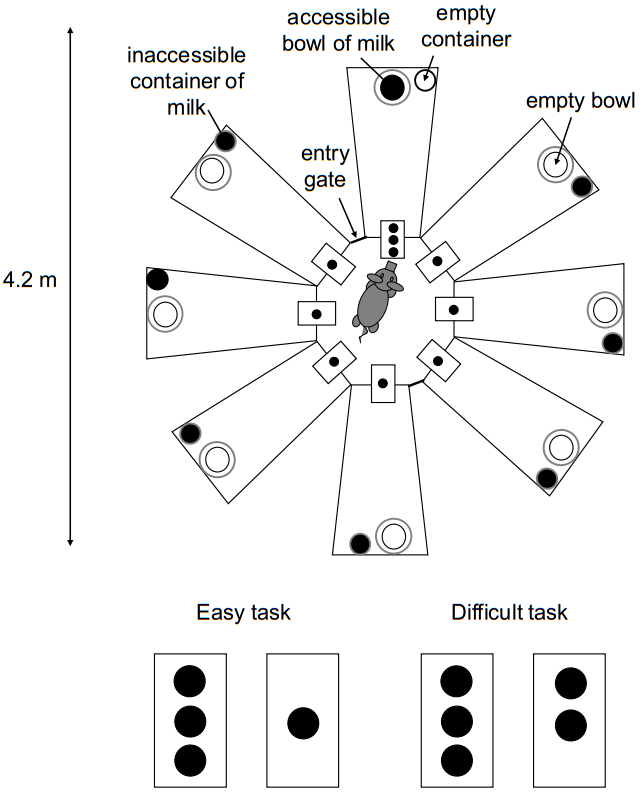


**Fig. S1 Schematic diagram of the learning area within the 8-arm radial maze and the visual cues used for the easy and difficult learning tasks**. Figure modified from Wang et al. 2007 Am J Clin Nutr

**Fig. S2**

**a**


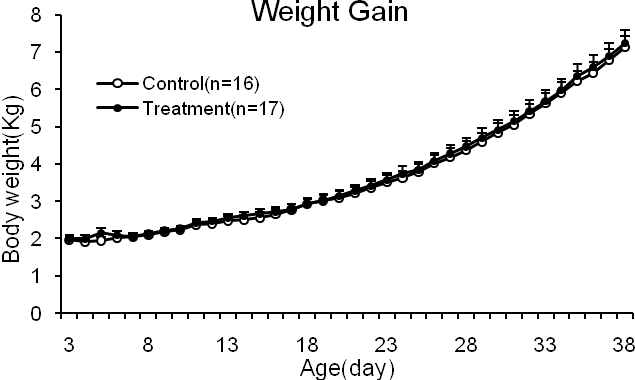


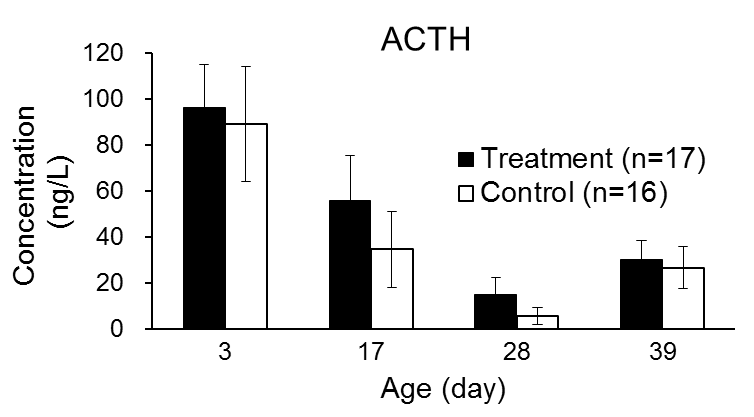


**b**

**
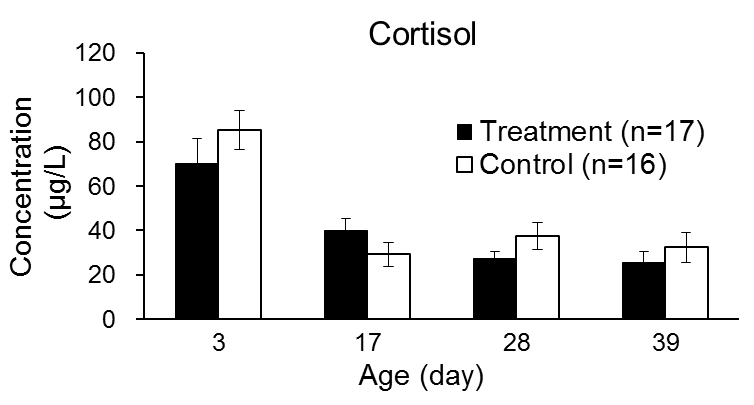
**

**c**

**Fig. S2 Body weight gain (a) and plasma hormone levels of ACTH (b) and cortisol (c) in the Lf and control groups during the study period.** No significant differences were found between the Lf and control groups (*p* > 0.05, Students’ *t* test). Data are expressed as mean ± SEM

**Supplementary Tables**

**Table S1** Concentration of lactoferrin, sialic acid and iron in the experimental diets

| **Diet** | Lactoferrin (mg/l) | | Sia (mg/l) | | |  |
| --- | --- | --- | --- | --- | --- | --- |
|  | Calculated LF | Analyzed Lf*^1^* | Analyzed Sia in Lf*^2^* | Sia theoretical Lf +basal | Analyzed Sia*^2^* | Iron*^3^* (mg/Kg) |
| Control | 50 | 57 | 0.29 | 125 | 125 | 138.15 |
| Treatment | 550 | 597 | 3.19 | 128 | 134 | 108.65 |

*^1^* Enzyme linked immunosorbent assay (ELISA) method (E11-126, Bethyl)

*^2^* High-performance liquid chromatography (HPLC) method (Agilent 1200, Agilent Technologies)

*^3^* Inductively Coupled Plasma-Optical Emission Spectrometer (ICP-OES) method (720-ES, Varian)

**Table S2** Primer sequences and accession number used in real-time PCR experiments for BDNF

| **Gene Name*^1^*** | **Accession Number** | **Primer Sequences** | **Products size (bp)** |
| --- | --- | --- | --- |
| **BDNF** | NM_214295 | F: TCTACGAGACCAAGTGCAATCCTAT | 73 |
|  |  | R: TTCCAGTGCCTCTTGTCTATGC |  |
| **GAPDH** | NM_001206359 | F: GGAAGCTTGTCATCAATGGAAAGG | 79 |
|  |  | R: ACCAGCATCACCCCATTTGA |  |
| **HPRT1** | NM_001032376 | F: GCCGAGGATTTGGAAAAGGTTTTTAT | 91 |
|  |  | R: CCTCCCATCTCTTTCATCACATCTC |  |

*^1^* BDNF: brain-derived neurotrophic factor; GAPDH: glyceraldehyde-3-phosphate dehydrogenase; HPRT1: hypoxanthine phosphoribosyltransferase 1
